# Effect of occlusal anatomy of CAD/CAM feldspathic posterior crowns in the stress concentration and fracture load

**DOI:** 10.1002/cre2.454

**Published:** 2021-07-09

**Authors:** Alexandre Luiz Souto Borges, João Paulo Mendes Tribst, Aline Lins de Lima, Amanda Maria de Oliveira Dal Piva, Mutlu Özcan

**Affiliations:** ^1^ Department of Dental Materials and Prosthetics São Paulo State University (UNESP), Institute of Science and Technology São José dos Campos Brazil; ^2^ Division of Dental Biomaterials, Center for Dental and Oral Medicine, Clinic for Reconstructive Dentistry University of Zurich Zürich Switzerland

**Keywords:** CAD/CAM, crown, finite element analysis, mandibular molar, stress

## Abstract

**Objectives:**

This study evaluated the effect of restoration occlusal design on the maximum fracture load and stress distribution of a feldspathic ceramic crown.

**Materials and Methods:**

Twenty dentin analogues were used to simulate a full‐crown preparation. Next, 20 feldspathic crowns were milled according to the occlusal design parameter available in the CAD database (Young or Adult). The crowns were cemented with dual cure resin‐cement and loaded until fracture at 1 mm/min crosshead speed. Data were analyzed by using one‐way ANOVA and Tukey tests (*p* < 0.05). The same geometry and experimental setup was modeled and exported to the computer aided engineering software and tensile stress concentration was calculated using the finite element method with 300 N occlusal load simulation.

**Results:**

The occlusal anatomy significantly influenced the load‐to‐fracture (*p* < 0.05). Adult design showed higher mean values (1149 ± 201 N) than Young design (454 ± 77 N). The maximum principal stress criteria showed similar stress pattern for both designs, however, the highest stress concentration was calculated for Young design (91 MPa) in the occlusal surface.

**Conclusions:**

An anatomy design with reduced cusp angulation and less evident occlusal sulcus can reduce the stress concentration and increase the fracture load for feldspathic CAD/CAM posterior crowns.

## 1 INTRODUCTION

Computer‐aided design and computer‐aided manufacturing (CAD‐CAM) technology allows the manufacture of indirect restorations with adequate dimensional accuracy, which is a key factor that influences the restoration longevity (Wang & Sun, 2020).

In a digital workflow, the ceramic materials stand out due to the superior aesthetic appearance, biocompatibility, durability, mechanical properties, and resistance to coloration in comparison to composite resins. However, ceramics are structurally more brittle, that is, more prone to fail (Zimmermann et al., 2017).

Feldspathic ceramics have been strengthened with alumina addition (50%) to their contents and is one of the ceramics commonly used for CAD/CAM. It consists of a fine grained feldspathic ceramic compacted into a block. Beyond Glass‐based dental ceramics have excellent aesthetic and mechanical properties, it needs an adequate surface treatment and luting procedure to achieve the highest bond strength and longevity (Moura et al., 2020).

For posterior crowns, the use of feldspathic ceramic provides a 12‐year survival estimate in 95% on molars and 94.7% on premolars being suggested as a very acceptable result (Otto & Mörmann, 2015). The stress concentration for posterior crowns with adequate thickness using these materials also present adequate magnitude (Dal Piva et al., 2018).

The CAD/CAM restoration mechanical response can be affected by crown design, tooth preparation, cementation and material thickness (Zimmermann et al., 2017). Regarding the design, a factor that could be strongly linked to fractures of dental restorations is their occlusal anatomy. For ceramic crowns, the cusp angles can influence the fracture resistance (Bowley et al., 2013) and the stress distribution (Liu et al., 2014). However, the occlusal anatomy and the presence of an occlusal sulcus still need to be verified (Sornsuwan & Swain, 2011). Since, crowns with thicker margins, smaller cusp angle and totally bonded are recommended to reduce the prosthesis failure susceptibility (Zhang et al., 2016). This can be justified because a lower crown thickness and higher cusp angle promotes a lower crack initiation load (Shahmoradi et al., 2020). However, these information are not available for feldspathic ceramic CAD/CAM crowns. Based on this, the aim of the current study was to investigate the influence of different occlusal anatomies on the fracture load and stress distribution of feldspathic ceramic full‐crown restorations.

## 2 MATERIALS AND METHODS

Ethical approval and informed consent was not sought for the present study because this article does not contain any studies with human or animal subjects.

Twenty (20) crown preparations were machined in epoxy resin (G10, Protec, São Paulo, Brazil) using CAD/CAM (Computer Aided Design/Computer Aided Machine) technology. The occlusal and axial walls were uniformly reduced (1.5 mm). The preparations presented rounded internal angles, chamfer preparation, and total occlusal convergence of 20°. Polyether (Impregum F, 3M ESPE) was used to simulate the periodontal ligament. To standardize the simulated periodontal ligament layer, a uniform coverage of approximately 0.3 mm was obtained by immersing the specimen's root portion in plastic wax (PW 1 Plastic—Kota Imports, São Paulo, SP, Brazil) for 2 s up to the marked distance of 1.5 mm. The constant flow of the wax was obtained by means of an electric wax heating apparatus (Mega Bell, Cera Matic Júnior, São Paulo, Brazil) with temperature control at 90°C confirmed by a thermometer. Then, the abutments embedded in self‐cured polyurethane resin (25 mm Ø × 20 mm height) up to 1.5 mm bellow the chamfer (Dal Piva et al., 2019). After the resin polymerization, the set was immersed in water at 75°C for 1 min to remove the wax layer, cleaned in an ultrasonic bath with distilled water for 5 min, then the polyether was applied around the root surfaces and finally the abutments were inserted in the resin (25 mm Ø × 20 mm height) up to 1.5 mm bellow the chamfer (Dal Piva et al., 2019).

An epoxy resin filled with glass fibers is a commonly used as a dentin‐like material since 2010 due to its similar elastic and adhesive properties to wet dentin (Kelly et al., 2010). According to the authors, the woven glass‐fiber‐filled epoxy (G10) can be compared to human dentin tissue bond strength. This material also demonstrates sufficient compatibility, in terms of both bonding and elastic behavior, to be used as dentin substitute to support ceramic specimens for in vitro tests (Dal Piva et al., 2019).

**FIGURE 1:**
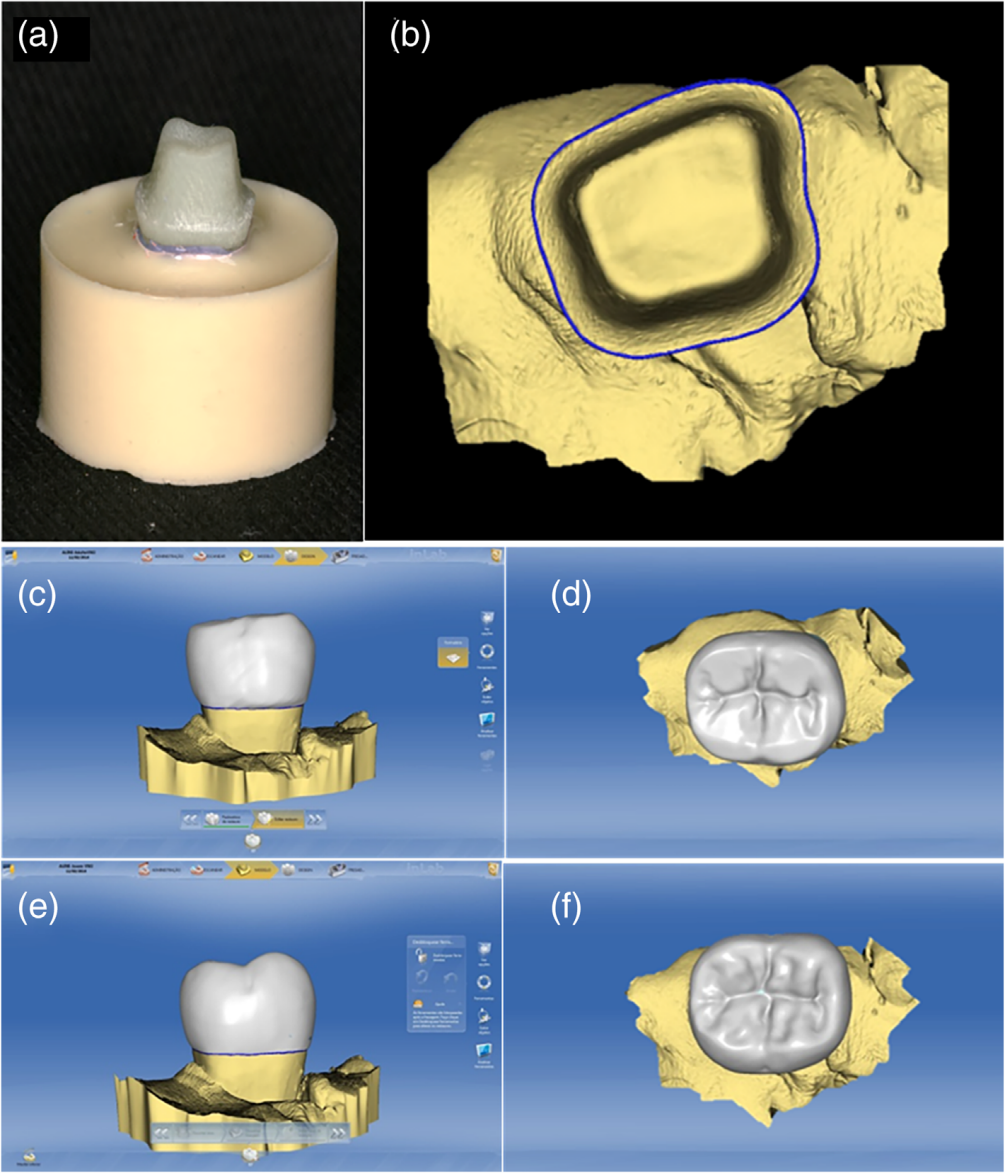
(a) substrate preparation, (b) digital impression, (c) adult design buccal view, (d) adult design occlusal view, (e) young design buccal view and (f) young design occlusal view

A thin layer of titanium dioxide‐based powder (Ivoclar Vivadent, Schaan, Liechtenstein) was sprayed onto each abutment for scanning (inEos Blue, inLab SW4.2, Sirona, Bensheim, Germany) and subsequent crown design. For that, the Cerec inLab (5884742 D329, Sirona for Dental Systems, Bensheim, Germany) database was used to provide the restoration anatomy information, and two shapes of crowns were obtained: “Adult”’ (AD—plane cusp) and “Young” (Y—high cusp heights; Figure 1). The crowns were randomly allocated in groups according to the anatomy shape (*n* = 10).

The G10 preparations were etched with 10% hydrofluoric acid (HF) for 60 s, washed with air/water jet for 30 s, and then dried (Tribst, Monteiro, et al., 2019). Before the cementation, a thin layer of adhesive (Single Bond Universal, 3M ESPE) was applied. The intaglio surface of each crown was air‐abraded with 50 μm alumina particles at a pressure of 20 bar from a 10 mm distance for 50 s with a constant particle incidence angle at 45°.

**FIGURE 2:**
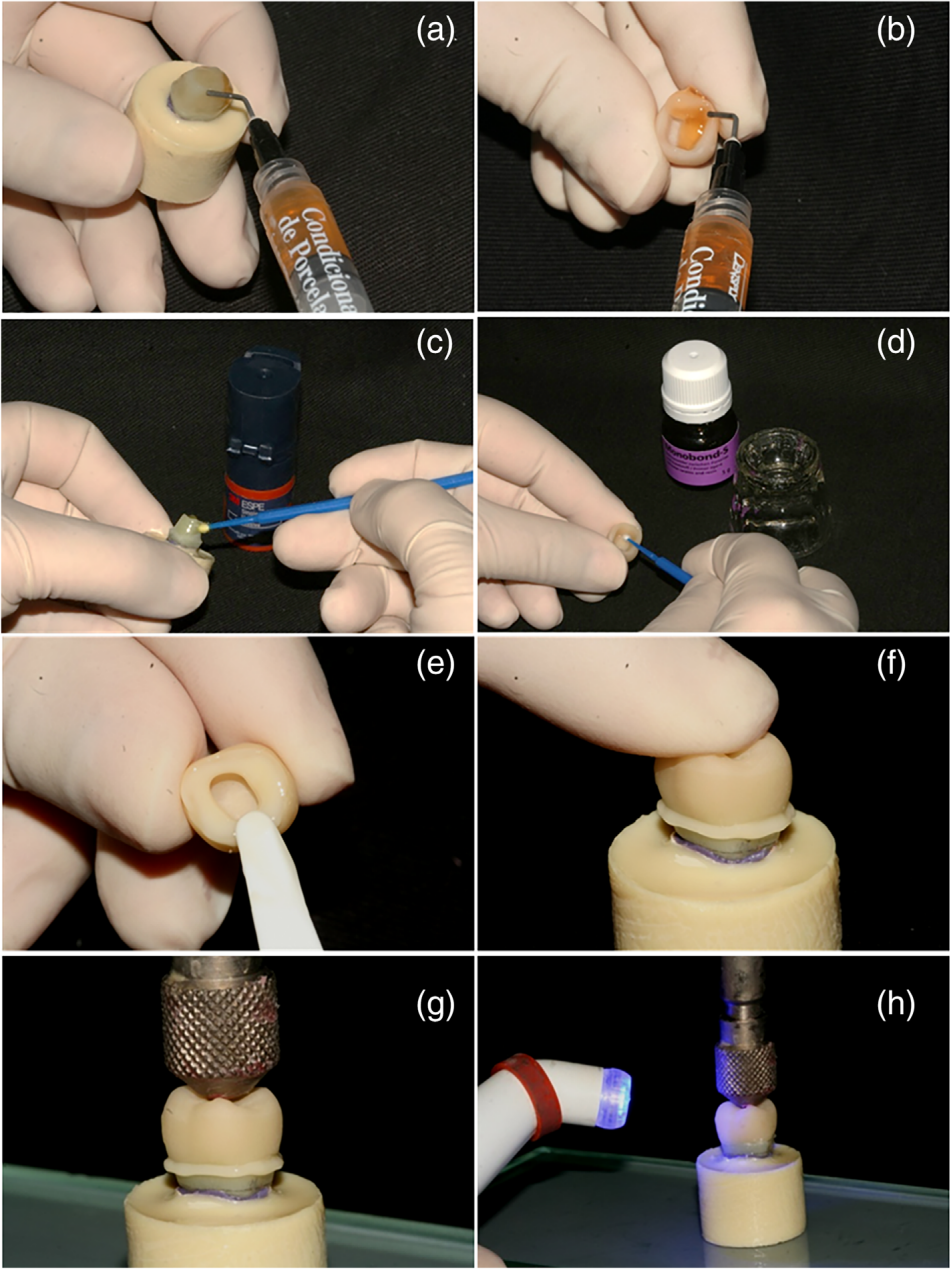
(a) substrate etching, (b) crown etching, (c) adhesive application, (d) silane application, (e) cement around the restoration margins, (f) digital pressure, (g) weight pressure and (h) light curing

The cement (RelyX ARC, 3M ESPE), was manipulated in 1:1 ratio and applied onto the intaglio surface of the crowns, which were then placed on the respective abutment and kept under a 750 g weight for 5 min. The excess resin‐cement was carefully removed and light‐cured (Radii‐cal LED curing light, SDI; 1200 mW/cm^2^) for 20 s on each restoration surface. The cemented restorations were stored in 37°C distilled water for 24 h before the fracture test (Figure 2).

Each crown was submitted to a single load to fracture test (load cell of 1000 kgf and 0.5 mm/min of cross‐head speed) in a universal testing machine (DL‐1000, EMIC, São José dos Pinhais, Brazil). The data was recorded as the mean load value (in N) of both groups. The maximum load to fracture data were statistically analyzed using one‐way analysis of variance (ANOVA) and the post‐hoc Tukey multiple range tests (*α* = 0.05; Tribst, Dal Piva, et al., 2019).

A three‐dimensional (3D) model of an upper first molar was used to evaluate the tensile stress of the resin crown. The model containing root, pulp chamber, periodontal ligament and fixation cylinder was imported to Rhinoceros CAD software (version 5.0 SR8, McNeel North America, Seattle, WA). For each group (Young and Adult), the anatomy previous provided by the database during the specimens preparation was exported as STL file to the CAD software and converted to NURBS using a reverse engineering plugin.

**FIGURE 3:**
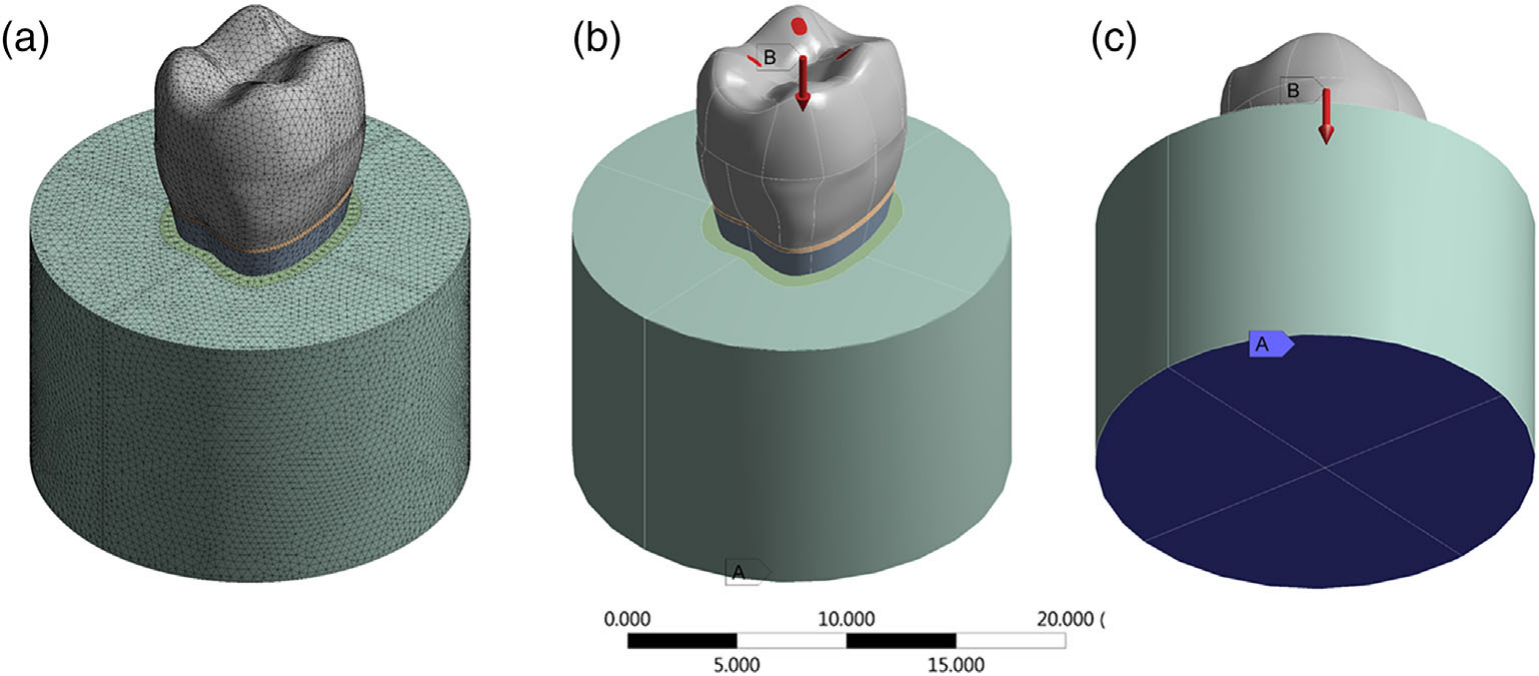
(a) Meshing for the finite element analysis, (b) load application and (c) fixed support

The volumetric solids were imported to the analysis software (ANSYS 16.0, ANSYS Inc., Houston, TX) in STEP (STandard for the Exchange of Product model data) format. A mesh convergence test (10%) determined 331,538 nodes and 223,955 tetrahedral elements for the Young design; and 325,224 nodes and 197,852 elements for the Adult design preparation (Figure 3).

The contacts were considered perfectly bonded. The structural mechanical analysis was performed loading the groups with 300 N distributed at contact areas, while the base was constrained following the experimental setup (Penteado et al., 2019). The materials properties simulated in the present study are summarized in Table 1. The maximum principal stress (MPS) criterion was used to analyze the tensile stress concentration. Results were reported in stress maps and the peaks were recorded.

**TABLE 1:** Material properties simulated for the finite element analysis

Material	Young's modulus (GPa)	Poisson's ratio
Polyurethane	3.60	0.30
Polyether	0.05	0.45
Dentin analogue	14.90	0.32
Cement	5.50	0.30
Acrylic resin	6.00	0.30

*Note*: Owner characterization was done during a pilot study.

## 3 RESULTS

One‐way ANOVA showed that the crown design influenced on the fracture load (*F* = 104.17; *p* = <0.001). For Adult design the load to fracture average was 1149 ± 201 N and for Young design, the load to fracture average was 454 ± 77 N.

**FIGURE 4:**
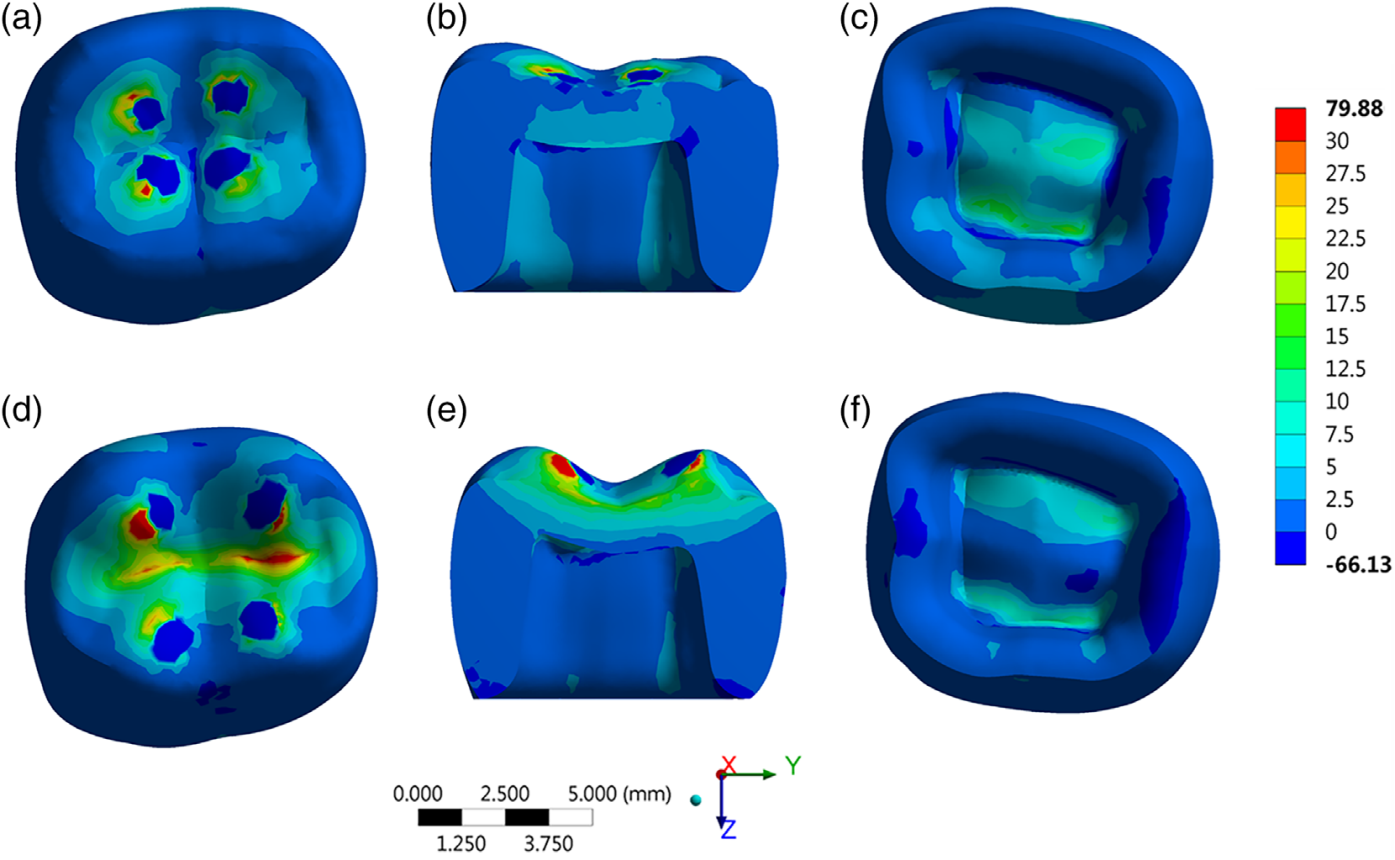
Stress maps for Young design (a–c) and adult design (d–f)

The Young design concentrated more stress compared to Adult design restorations (Figure 4). The higher the cusp angle, the higher the stress concentration in the crown's intaglio surface and occlusal surface. The peak of tensile stress was 91 MPa for Young design and 39 MPa for Adult design. The stress concentration regions for both groups suggest that the occlusal fissures would be the failure origin.

## 4 DISCUSSION

The goal of this study was to evaluate the effect of CAD/CAM feldspathic crowns occlusal anatomy on the fracture load and stress concentration. It was observed that the design influenced the crowns' fracture load and stress concentration, leading to the rejection of the study's hypothesis.

Ceramic crowns should present a sufficient resistance against the maximum chewing forces. The average bite force for man was reported as 587 N and 424 N for woman (Calderon et al., 2006). This force magnitude can induce early failures for the Young design bases in the fracture resistance calculated for this group. A previous study reported 634 N as an average maximum fracture load for 1.5 mm feldspathic crowns (similar to the present study), and, the authors did not indicate this material for posterior crowns (Zimmermann et al., 2017). However, the present study complement this findings since the crown's Adult design showed an adequate fracture load to resist against the bite force. This can explain the good clinical performance in a 12‐year clinical study using this material for posterior crowns (Otto & Mörmann, 2015).

A previous study reported that the performance of feldspathic crowns was superior to bilayer crowns in the fatigue test, justifying the premature fractures of bilayers due to the weakness in the core/veneer bond (Zahran et al., 2008). At the same time, the use of CAD/CAM and monolithic crowns can reduce the presence of flaws and critical defects. Clinically, all‐ceramic restorations commonly fail through slow crack growth resulting from fatigue caused by masticatory stresses (Zahran et al., 2008). The absence of fatigue in the present study is a limitation, however, the initial fracture load can assist further studies in select the suitable anatomy prior to the milling procedure.

An in vitro study reported that a feldspathic ceramic had the best fatigue behavior when cemented to a dentin‐like substrate in comparison to lithium disilicate, polymer infiltrated ceramic and a nanohybrid composite resin (Weitzel et al., 2020). Also, they reported that a complete ceramic crown could result in a different stress scenario and may lead to different results from those that they obtained in their study (44 MPa). However, for the Adult design, the stress magnitude can be considerable relatively closer (39 MPa). The stress pattern with the highest peak at the occlusal region is already reported in molar crowns as a natural behavior for the load dissipation (Oladapo et al., 2018).

Another study reported that the loads at initial radial cracks in feldspathic disc‐shaped specimens had overall mean equal to 534.83 N (Guilardi et al., 2020). The authors used 1 mm thickness ceramic restorations, what is a more conservative approach in terms of tooth preparation, however, does not allow a direct comparison with the present study (1.5 mm).

An important clinical step is the luting procedure. The adhesive cementation can increase the strength of weak ceramics and it is recommended for leucite glass ceramic and feldspathic ceramic crowns (Bindl et al., 2006). For that reason, the present study used a resinous adhesive cement during the samples preparation.

The fracture load calculated in a previous report for the same material (Mk II, Vita Zahnfabrik, Germany) was 985 N what can be comparable with the fracture load calculated in the present study for the Adult design (1149 ± 201 N). This can be justified because the authors used a flat occlusal design for the experimental design, which is more similar to a low cusp design as the adult parameter.

A previous study compared different surface treatments for premolar crowns using feldspathic ceramic. The authors reported that the specimens reacted against the applied compressive load as a single unit showing higher fracture load than the reported mean maximum masticatory forces (Ibraheim, 2019). A similar finding for molars can be performed in the present study, however, only for the Adult design.

Despite the fitting accuracy of CAD/CAM crowns, an ideal occlusal surface is hard to design, since it requires the proper registration of the dynamic relation between maxilla and jaw, the occlusion and the patient individualization (Muric et al., 2019). In this sense, the use of Young morphology should not be indicated for feldspathic ceramic crowns since it present higher stress concentration and lower load to fracture values, suggesting a higher failure possibility.

The literature reports that full‐crowns designed with dental database but considering the antagonist arch had a significantly smaller occlusal vertical discrepancy than those made with only the correlation design technique (Fasbinder & Poticny, 2010). The present study complement this report suggesting that the ceramic crown anatomy should be based on the patient's occlusion and restorative material fracture resistance and not only based on the database anatomy.

The major difference between both crown designs simulated in the present study were the cusp angle and the occlusal sulcus. A previous study evaluated the fracture load of posterior ceramic crowns with and without post‐milling manual enhancement of occlusal morphology, which can be comparable, respectively, with the more detailed anatomy (Young) and the less detailed anatomy (Adult), evaluated in the present study. The authors concluded that this procedure should be considered detrimental for monolithic CAD/CAM‐generated crowns and should be avoided (Passos et al., 2018). The present study corroborates with this statement since the Young design correspond to a restoration more prone to fail. This behavior can be explained because the bite force loads can generate a horizontal force component which tends to open up the fissure space and create tensile stress concentration and subsequent cracks at the base of the fissure (Wan et al., 2019).

A previous finite element study suggest that if the cusp angle increases, a higher oblique load incidence will also happen for a premolar restored with ceramic endocrowns (da Fonseca et al., 2018). This concern can also affect molar crowns as reported in the present study, considering only the fracture load and the stress concentration for feldspathic crowns.

It is related that a surface irregularity can be considered as a discontinuity in the material's microstructure; thus, it can start a critical crack in the crown, reducing the restoration longevity (Shahmoradi et al., 2020). Therefore, a simplified anatomy using an Adult design can be positive to avoid the presence of critical regions. Simplify the tooth occlusal anatomy can reduce the patient's muscular activity and masticatory efficiency as ported for artificial tooth for example (Türp et al., 2008). However, the effect of one tooth restored with a full‐crown designed with simplified anatomy in patients with adjacent natural dentition has not been reported yet.

As this study's limitations, is important to emphasize that a load to fracture test does not replicate all the clinical loads to which the restoration can be exposed. The thermoaging also can reduce the bond strength between the restoration and cement and reduce the failure load values (Tribst et al., 2020). The isotropic material simulated in the finite element study is a simplification and cannot be found in the clinic, also different patient's occlusion can influence the load incidence pattern and modify the biomechanical response (da Fonseca et al., 2018).

## 5 CONCLUSION

Based on the results, the following conclusion can be drawn:

An anatomy design with reduced cusp angulation and less evident occlusal sulcus can reduce the stress concentration and increase the fracture load for feldspathic CAD/CAM posterior crowns.

## CONFLICT OF INTEREST

The authors declare that they have no conflict of interest.

## AUTHOR CONTRIBUTIONS

All authors have contributed to the study design and substantially contributed to the drafting of the manuscript. Aline Lins de Lima and Amanda Maria de Oliveira Dal Piva substantially contributed in analysis and interpretation of data. Alexandre Luiz Souto Borges and João Paulo Mendes Tribst contributed to acquisition of data. All authors contributed to the preparation and critical revision of the manuscript, and agree to be accountable for all aspects of the study.

## Data Availability

Data available on request from the authors.
